# Psychological Factors Associated with General Quality of Life in the Context of COVID-19 Pandemic: A Cross-Sectional Study on a Multicultural Sample of Romanian Medical Students

**DOI:** 10.3390/healthcare12131243

**Published:** 2024-06-21

**Authors:** Alexandra Ioana Mihăilescu, Ovidiu Popa-Velea, Adela Magdalena Ciobanu, Liliana Veronica Diaconescu, Alexandra Graur, Ioana Ioniţă, Mara Carsote

**Affiliations:** 1 Department of Medical Psychology, Faculty of Medicine, “Carol Davila” University of Medicine and Pharmacy, 020021 Bucharest, Romania; alexandra.mihailescu@umfcd.ro (A.I.M.); liliana.diaconescu@umfcd.ro (L.V.D.); ioana.ionita@drd.umfcd.ro (I.I.); 2 Department of Psychiatry, “Prof. Dr. Alexandru Obregia” Clinical Hospital of Psychiatry, 041914 Bucharest, Romania; adela.ciobanu@umfcd.ro (A.M.C.); graur.alexa@gmail.com (A.G.); 3 Neuroscience Department, Discipline of Psychiatry, Faculty of Medicine, “Carol Davila” University of Medicine and Pharmacy, 020021 Bucharest, Romania; 4 Department of Endocrinology, “Carol Davila” University of Medicine and Pharmacy, 020021 Bucharest, Romania; carsote_m@hotmail.com; 5 Department of Clinical Endocrinology V, “C.I. Parhon” National Institute of Endocrinology, 011863 Bucharest, Romania

**Keywords:** COVID-19, quality of life, cultural, personality, stress, fear, medicine, students

## Abstract

The COVID-19 pandemic had a significant impact on the general quality of life (GQOL) of a large number of individuals, including those in the academic environment. This study investigated GQOL in a sample of 613 Romanian medicine students (81.57% were female; mean age = 21.40 ± 1.749 years) in relation to their Big Five personality characteristics, Perceived Stress and Fear of COVID-19. The study was conducted between June 2020 and March 2022. These variables were investigated with the Big Five Inventory-2: Extra-Short Form, the Fear of COVID-19 Scale (FCV-19S), the Perceived Stress Scale-10 and the Satisfaction with Life Scale (SWLS). Statistical analysis included hierarchical linear regression and *t*-tests. The results indicated a significant direct relationship between GQOL and the personality traits of Conscientiousness, Extraversion and Agreeableness. However, a significant inverse relationship was observed between GQOL and Perceived Stress and Neuroticism. Fear of COVID-19 was significantly higher in women, while no other socio-demographic variables were associated with GQOL. A total of 61.7% of the studied population returned to their original residency during the pandemic years. These results could be important for better understanding the vulnerability to significant epidemiological events in academic populations and for planning adequate preventive or interventional measures.

## 1. Introduction

The Coronavirus Disease 2019 (COVID-19) pandemic has been considered an unprecedented challenge to public health systems around the world, impacting the lives of a large number of individuals. In order to diminish the number of new cases and fatalities, energic measures of containment were necessary and applicable to a large territory. The sequels of these measures, including psychological suffering and its ripple effects, have been considered, to a certain point, unavoidable. Nevertheless, during and in the aftermath of the pandemic, the significant effects of social isolation, the disruption of routine activities, the restriction of movements, the decline in work habits and income and the lack of access to medical care have proven to represent a high cost for many individuals and countries around the world [1,2,3]. Romania was no exception, being heavily affected by the pandemic [4,5]. Notably, at the beginning of June 2020, almost 20,000 cases of coronavirus infection were registered, while in March 2022, this number increased to approximately 2,750,000 total cases [6].

A significant consequence of the COVID-19 pandemic, reported worldwide, was the global deterioration in mental health, reflected in an increased prevalence of distress, sleep disorders, generalized anxiety, post-traumatic stress disorder (PTSD) and depression [7,8,9,10,11,12].

Even in individuals who did not become ill, the pandemic created the premises for suffering, both in the short and long term. In this sense, one of the key variables that significantly deteriorated during the pandemic [13,14,15,16] was the global quality of life (GQOL). According to existing literature, this effect was caused by a series of trait and state variables:

- Among the trait variables (i.e., those “relatively stable pre-existing characteristics an individual brings to a situation”) [17], the Big Five model personality traits [18] may play a key role. In this regard, prior studies focused on personality traits during the pandemic found, for example, that Openness has been associated with mixed results in terms of COVID-19 guideline adherence [19,20,21]. Conscientiousness has been a positive predictor of higher adherence, prevention use [22,23,24] and a negative predictor of depression [25]. Oppositely, Extraversion has been associated with reluctance to adhere to social distancing measures, a series of hazardous behaviors, low adherence, medical sufficiency [26,27] and negative perceptions, when combined with neuroticism [27]. Agreeableness has been described as negatively associated with stress, anxiety and as being a positive predictor of preventive behavior and confidence in medicine [28]. Neuroticism has been correlated with higher stress and increased prevalence of anxiety and depression [29,30] and with mixed associations with guidance adherence [20,31];
- Among the state variables (i.e., those “influencing behavior over a relatively short time frame”) [17], Fear of COVID-19 was demonstrated to decrease GQOL but was equally connected to many additional mental health problems, such as anxiety, insomnia and depression [32,33,34]. In turn, the amount of Perceived Stress during the pandemic has significantly increased and generally lowered GQOL [35] via decreases in physical activity level, changes in nutrition, increased alcohol consumption, smoking and the impact on pre-pandemic daily habits [36,37,38,39,40]. An additional burden was caused by the detection of new medical and surgical entities that were directly related to the viral infection or to the associated restrictions, including social and medical (such as access to healthcare check-up, particularly during the first pandemic waves). Interestingly, the literature also suggests the possibility of a direct connection between stress and improved lifestyle behaviors, leading to the preservation of GQOL. This perspective posits that heightened stress awareness during the pandemic may motivate individuals to adopt a healthier lifestyle [35], thereby mitigating the negative impact of stress on GQOL.

In academic settings, the abovementioned findings and relationships, although examined, are, to a certain point, unknown, making this topic interesting for research. Furthermore, these kinds of studies are important, given the fact that the academic population was one of the most affected social groups during the pandemic. Significant risks derived from exposure to repeated lockdowns, uncertainty and restrictions all interfered with the students’ general ability to learn and interact [41]. GQOL during the pandemic could have also been shaped by cultural differences, reflected in the variety of cultural norms, social positions, access to resources and healthcare facilities. Also, the change in the essence of students’ academic interactions from direct ones to indirect (mostly online) ones significantly increased psychological morbidity [42,43,44]. In this respect, many students exhibited depressive symptomatology, anxiety and sleep disturbances [45,46,47,48].

These damaging effects are especially relevant among medicine students, as they may cumulate the specific demands that they faced during the pandemic with previously higher than average stress load, elevated rates of depression, anxiety and burnout [49], with durable consequences for their general adjustment and for their psychological and physical health [50]. During the pandemic, they had not only to adjust themselves to distance/online learning (similar to students from other fields), but at the same time, they took part in the care of patients with COVID-19.

This study aimed to respond to the following research questions, with reference to the specific context of the COVID-19 pandemic:

- What is the impact of socio-demographic factors on medical students’ GQOL?
- What is the relationship between personality traits assessed dimensionally and medical students’ GQOL?
- How does Fear of COVID-19 and Perceived Stress impact medical students’ GQOL?

Specifically, we formulated the following research hypotheses:

**Hypothesis 1.**  *Socio-demographic factors (age, residential status and gender) could represent independent predictors of GQOL.*

**Hypothesis 2.**  *Personality traits (Openness (O), Conscientiousness (C), Extraversion (E), Agreeableness (A) and Neuroticism (N)) are GOQL predictors. O, C, E and A are positive predictors, while N is a negative predictor.*

**Hypothesis 3.**  *Fear of COVID-19 is a negative GQOL predictor.*

**Hypothesis 4.**  *Perceived Stress is a negative GQOL predictor.*

## 2. Materials and Methods

### 2.1. Study Design and Procedure

The design of the study was cross-sectional, with participants recruited through convenience sampling via the internet. This allowed an efficient data collection from a diverse population of medical students exposed to social distancing measures within the study time frame.

The study was conducted between June 2020 and March 2022. The study protocol involved the administering of an online set of questions containing the study instruments, which were distributed through the social media groups of the university.

### 2.2. Participants

The participants were undergraduate medicine students undergoing their training at the “Carol Davila” University of Medicine and Pharmacy, Bucharest, Romania (CDUMP). This university (located in the capital of Romania) is the largest medicine school in Romania, with an approximate population of students of 6000 and gathering students from many regions of the country, thereby offering a multicultural sample of an Eastern European country.

The inclusion criteria were (a) age 18 years or older; (b) having the status of current undergraduate student in the abovementioned institution; and (c) having experienced lockdown, social distancing measures and isolation during the COVID-19 pandemic for at least three months. The exclusion criteria were represented by current self-reported somatic or psychiatric morbidity, chronic alcohol or drug use, cognitive deficits or any other impairment, which would render the understanding and completion of the study questionnaires difficult, and lack of completion of one or more study instruments.

The sample size was calculated according to the guidelines in the field [51]. The minimum sample size taken into consideration was 601 students, allowing for a confidence interval of 95% and a margin of error of ±4%.

From the total of 721 students who agreed to participate in the study, 108 students were removed because they did not meet the inclusion criteria and/or met the exclusion criteria, rendering a total of 613 subjects finally enrolled [113 (18.43%) men and 500 (81.57%) women]. The skewed distribution of the sample reflects the asymmetry in the gender distribution within the Faculty of Medicine, which is characterized by a significantly larger number of female students. The mean age was 21.40 (SD = 1.749). This sample was checked for representativity in terms of the balance between the number of students in preclinical/clinical tracks, resulting in no significant differences (1.08:1 vs. 1:1.04, *p* < 0.24).

Before being able to participate in this research, all participants received a brief explanatory statement about the study and completed informed consent forms. The study was conducted in accordance with the World Medical Association Declaration of Helsinki and was approved by the CDUMP Ethics Committee (No. 10097, approved on 13 May 2020). A researcher (AM) was available via email in case there were questions related to the process of filling the questionnaires.

### 2.3. Instruments

#### 2.3.1. Big Five Inventory-2: Extra-Short Form (BFI-2 XS) [52]

Each participant’s personality profile was investigated using a translated version of the Big Five Inventory-2: Extra-Short Form. This comprises a 15-item scale on the dimensions of personality (Extraversion, Agreeableness, Conscientiousness, Neuroticism and Openness to Experience). The answers are provided on a 5-point Likert scale (from 1—Strongly disagree to 5—Strongly agree). Data literature shows that this instrument displays good reliability (of approximately 80%) and good external validity, allowing its administration to a large variety of respondents [53,54,55]. In this sense, previous research has shown good indicators of cross-cultural generalizability [56].

#### 2.3.2. The Fear of COVID-19 Scale (FCV-19S) [57]

Fear of COVID-19 was investigated using a standardized, translated, 7-item scale [53], comprising both direct and indirect questions. Items were rated on a 5-point Likert scale (from 1—Strongly disagree to 5—Strongly agree), according to the respondents’ opinions on how well the item applied to them. All scores were summed up, with the total score reflecting the degree of fear the respondent felt (a higher score corresponded to a greater degree of fear). The scale has strong internal consistency reliability (Cronbach alpha: 0.87), which is consistent with prior studies on the scale in a range of other national groups, including Romanian individuals (Cronbach value: 0.88) [58] and US college students (Cronbach alpha value: 0.80) [59]. The construct validity has been established through the observation of a positive correlation between FCV-19S scores and scores of Perceived Stress and life satisfaction [60,61].

#### 2.3.3. Perceived Stress Scale-10 [62]

This represents a standardized 10-item scale, where the answers are provided on a 4-grade Likert scale, ranging from 0—Never to 4—Extremely often, according to how frequently the respondent experienced the described circumstances in the last month. The total sum indicates the degree of stress experience, with a higher score pointing to higher distress. The Romanian version [63] displays a Cronbach alpha value of approximately 0.85, making it suitable for use in this study.

#### 2.3.4. The Satisfaction with Life Scale (SWLS) [64]

This instrument has five items, rated from 1 to 7 (from 1—Strongly disagree to 7—Strongly agree). The sum of scores is a measure of the degree of life satisfaction. The values are classified from 5–9 = Extremely dissatisfied to 31–35 = Extremely satisfied. The Cronbach alpha value of the test is around 0.80 [65]. The Romanian version of this scale [66] was included in this study due to its similar statistical power to other short quality of life scales [67] and its description in the literature as a scale able to measure the global quality of life (GQOL).

In addition to these instruments, the participants provided information about their age at the time of testing, study year and field of study (general medicine, pharmacy, dentistry, nursing, dental technology and midwifery). The survey also collected information about their residential status and gender, allowing for a nuanced analysis of these variables’ impacts on GQOL (residential status was investigated by registering whether the person was able to return or not to their familiar living space (residence) during the pandemic).

### 2.4. Data Processing

All responses were processed anonymously, and a numerical code was assigned for each participant. The collected data were accessible exclusively to study researchers (AIM, OPV, AMC, LVD, AG, LB, II, MC). Regular didactic staff had no access to the distribution, collection or interpretation of questionnaires.

The interpretation of the questionnaires was performed independently by two researchers (AIM, II) and cross-checked for congruence afterward. The final results were included in a SPSS 21 (SPSS Inc., Chicago, IL, USA) database.

The descriptive analysis included the computation of means and standard deviations of the demographic and psychological quantitative variables included in the study, while for qualitative variables, a distribution of frequency was realized.

The statistical analysis included a hierarchical linear regression performed to establish the comparative weight of GQOL predictors by grouping independent variables into 1. socio-demographic (age, gender, residence, study year), 2. psychological trait (personality) and 3. psychological state (Fear of COVID-19, Perceived Stress). The gender differences were explored through *t*-tests for independent samples. Throughout the statistical analyses, missing data were handled through list wise deletion. The assumptions of data normality and linearity were confirmed using histograms and partial plots. The independence of errors was checked through the Durbin–Watson test. For all calculations, the threshold of statistical significance was *p* < 0.05.

## 3. Results

### 3.1. Descriptive Analysis

Table 1 displays the summary of the study participants’ socio-demographic data.

The synthesis of data regarding the psychosocial variables in the whole sample is displayed in Table 2.

By gender, the descending hierarchy of the BFI-2 XS scores was Openness (M = 11.06, SD = 2.300), Conscientiousness (M = 10.10, SD = 2.096), Extraversion (M = 9.87, SD = 2.332), Agreeableness (M = 9.77, SD = 1.973) and Neuroticism (M = 8.56, SD = 2.662) among men, and Openness (M = 10.85, SD = 2.000), Conscientiousness (M = 10.28, SD = 2.111), Agreeableness (M = 10.22, SD = 1.818), Neuroticism (M = 10.00, SD = 2.559) and Extraversion (M = 9.48, SD = 2.286) among women.

Regarding Perceived Stress, the respondents were classified into three subgroups of low (0–13), average (14–26) and high (27–40) level of stress, in line with the scale’s specifications [57]. The majority of them (*N* = 365; 59.54%) were classified as experiencing average levels of Perceived Stress, followed by high levels (*N* = 134; 21.86%) and low levels (*N* = 114; 18.59%). By gender, men had lower Perceived Stress (M = 17.61, SD = 7.035) than women (M = 20.84, SD = 7.022).

In terms of Fear of COVID-19, 156 students (25.44%) were placed below the Q1 quartile, 178 (29.03%) between Q1 and Q2, 117 (19.08%) between Q3 and Q4, and 162 (26.42%) above the Q4 quartile. By gender, men had lower Fear of COVID-19 (M = 12.25, SD = 4.564) than women (M = 14.43, SD = 5.162).

Finally, concerning the GQOL, 147 students (23.98%) were placed below the Q1 quartile, 146 (23.81%) between Q1 and Q2, 154 (25.12%) between Q2 and Q3, and 166 (27.08%) above Q4. By gender, men had a slightly higher GQOL (M = 26.37, SD = 5.214) than women (M = 26.25, SD = 5.589).

### 3.2. Statistical Analysis

#### 3.2.1. Socio-Demographic Variables and Global Quality of Life

*t*-tests were used to evaluate the association between socio-demographic dichotomous independent variables (gender, residence status) and GQOL.

GQOL was not significantly associated with gender only: t (1, 611) = 0.219 (*p* < 0.827). Concerning residence, the association was significant in men [t (1, 111) = 2.080, *p* < 0.04], who benefited the most from conserving their habitual space for living.

To compare the global quality of life between medical students in preclinical and clinical tracks, we conducted *t*-tests. The results indicated that there were no significant differences between the two groups in terms of their GQOL scores. This lack of significant difference was consistent when we further analyzed the data by gender.

#### 3.2.2. Psychological Variables and Global Quality of Life

##### Trait (Big Five Personality) Variables and GQOL

In order to examine the relationship between the Big Five personality dimensions and GQOL, a linear regression was performed, including each of the investigated dimensions. The results, displayed in Table 3, indicate that Conscientiousness, Extraversion, Agreeableness and Neuroticism emerged as important predictors of global quality of life among the studied population.

The model explained 17.9% of GQOL variance, with three dimensions (Conscientiousness, Extraversion and Agreeableness) positively associated with GQOL and one negatively associated (Neuroticism). In men, it explained 19.9% of the variance, with one dimension (Extraversion) positively associated and another negatively associated (Neuroticism). In women, it explained 17.7% of the variance, with three dimensions (Conscientiousness, Extraversion and Agreeableness) positively associated with GQOL and one negatively associated (Neuroticism).

##### State Variables and GQOL

1. Perceived Stress and GQOL

We found a significant reverse correlation between GQOL and Perceived Stress (r = −0.422, *p* < 0.001), encountered both in men (F = 8.552 (df 2), *p* < 0.001) and women (F = 47.721 (df 2), *p* < 0.001).

2. Fear of COVID-19 and GQOL

These variables were negatively and significantly correlated (Pearson’s correlation r = −0.124, *p* < 0.002). By gender, this relationship was only encountered in women (Pearson’s correlation r = −0.128, *p* < 0.004) and not in men (Pearson’s correlation r = −0.104, *p* < 0.273).

Significant differences were present between men and women in terms of Fear of COVID-19, as shown by a two-tailed *t*-test for independent samples: t (1, 611) = −4.16, *p* < 0.001, 95% confidence interval = −1.16 … −3.24. Thus, the null hypothesis that there was no difference in the mean value between these two groups was rejected.

##### Psychological Predictors of Global Quality of Life

A hierarchical regression analysis was run in order to identify the quantitative independent variables significantly associated with GQOL and their comparative importance. The analysis was conducted in three successive steps, each one of them adding a new variable, to observe their incremental impact on GQOL.

First step (Model 1): We investigated the impact of personality traits alone. A moderate relationship was noticed between these predictors and GQOL (R = 0.430). The proportion of GQOL variance explained by them was 17.7%.

The significant predictors in Model 1 included

- Conscientiousness (β = 0.162, t = 3.768, *p* = 0.001);
- Extraversion (β = 0.160, t = 3.736, *p* = 0.001);
- Agreeableness (β = 0.091, t = 2.211, *p* = 0.027);
- Neuroticism (β = −0.258, t = −6.045, *p* = 0.001).

Openness did not significantly predict GQOL (β = −0.012, t = −0.282, *p* = 0.778).

Second step (Model 2): In the analysis, we considered the Fear of COVID-19 and the personality traits. The relationship strength slightly increased (R = 0.432), while the proportion of GQOL variance remained the same.

This essentially means that the significant predictors in Model 2 were the same as in Model 1, with Fear of COVID-19 not exerting a significant impact (β = −0.039, t = −0.912, *p* = 0.362). Fear of COVID-19 by itself is not a significant GQOL predictor.

Final step (Model 3): In the analysis, we included Perceived Stress, personality traits and the Fear of COVID-19. The strength of the relationship increased significantly (R = 0.503). The proportion of GQOL variance also increased to 24.2%.

The significant predictors in Model 3 included

- Conscientiousness (β = 0.135, t = 3.244, *p* = 0.001);
- Extraversion (β = 0.152, t = 3.678, *p* = 0.001);
- Agreeableness (β = 0.109, t = 2.732, *p* = 0.007);
- Perceived Stress (β = −0.333, t = −6.590, *p* = 0.001).

Neuroticism, while significant in previous models, became non-significant when Perceived Stress was added (β = −0.071, t = −1.404, *p* = 0.161), while Openness and Fear of COVID-19 remained non-significant predictors.

Model 3 demonstrates that Perceived Stress is a significant negative predictor of GQOL, reducing the influence of Neuroticism when added to the model. The personality traits of Conscientiousness, Extraversion and Agreeableness remained significant positive predictors.

As a whole, the hierarchical regression analysis revealed that personality traits, specifically Conscientiousness, Extraversion and Agreeableness, along with Perceived Stress, were significant predictors of GQOL among medical students during the COVID-19 pandemic.

The impact of Neuroticism diminished when Perceived Stress was included, highlighting the significant role of stress management in influencing the quality of life. Fear of COVID-19 did not significantly influence GQOL in this sample. This study emphasizes the double importance of trait variables (personality) and state variables (Perceived Stress) in understanding GQOL among medical students.

Separate hierarchical regressions run for GQOL predictors—by gender, residence and study cycle—did not change the list of predictors identified when running this statistic on the whole sample.

## 4. Discussion

The present study sought to investigate the distinct associations between GQOL and individual (socio-demographic and psychological) characteristics among Romanian undergraduate medicine students during the first three years of the COVID-19 pandemic.

The results provide valuable insights into the socio-demographic correlates of global quality of life (GQOL). The observed correlation between GQOL and re-establishing previous residence, moderated by gender, highlights important considerations regarding the impact of internal migration on the quality of life, particularly among young adults. Additionally, the significant positive effect of returning to previous residential status among men emphasizes the importance of familiar environments in providing social support and resilience during these challenging times.

### 4.1. Socio-Demographic Correlates

Hypothesis 1 was partially verified. GQOL was independent of the age or gender of the respondents. However, GQOL was correlated to re-establishing previous residence, this phenomenon being described as frequent during the COVID-19 pandemic in young adults, and reflecting a veritable internal migration. Previous literature identifies as possible causes of this phenomenon the lack of social and economic support and the poor access to care [68]. In the particular case of medicine students, a significant part of them may have sought refuge in rural areas or returned to their hometowns for protection and assistance [69].

In our study, the GQOL–residence relationship was moderated by gender, with a significant positive effect of returning to previous residential status among men. They benefited the most from returning to their habitual living space, this possibly reflecting the secondary benefits stemming from this situation. Such gains may have included a heightened sense of mastery over the environment and a higher probability of delivering and receiving social support. A distinct contribution to the abovementioned effect could have also been brought by the origin of the respondents, who were in a large proportion from the southern and eastern parts of the country, where men traditionally have higher control over the relationships inside their families [70,71]. Nevertheless, further research is needed to explore the underlying mechanisms that make familiar environments more beneficial for men. This could include qualitative studies to understand the specific type of social support men give and receive in these settings and its perception.

The abovementioned associations are partially supported by prior data, pointing out a negative relationship between loneliness and intolerance to uncertainty (both mitigated by conserving one’s residence) and poor quality of life [72]. Oppositely, social connections and community ties may offer a sense of belonging, emotional support and resilience during difficult times, acting as an efficient coping mechanism [73]. At a societal level, our data indirectly represent a reflection of the solidarity during the COVID-19 pandemic, which has also been described in the literature [74].

### 4.2. Psychological Characteristics

Regarding personality characteristics, this sample displayed higher levels of Openness to Experience, Consciousness and Agreeableness and lower levels of Neuroticism and Extraversion. By gender, Extraversion exhibited the smallest scores in women, while Neuroticism presented the lowest scores in men. The investigation of the distinct associations of personality characteristics revealed a positive association of Conscientiousness, Extraversion and Agreeableness with GQOL.

Hypothesis 2 was partially verified. Conscientiousness, Extraversion and Agreeableness may together construct a cluster consistent with higher emotional stability, adherence to rules, striving to understand their meaning, ease in social interactions and greater perceived social support, all contributing to higher GQOL. In particular, Conscientiousness was found in the literature to diminish Perceived Stress in the context of a pandemic [75], with individuals with higher scores in this domain being easily compliant with protective measures like home confinement [23,76]. Extraversion was reported to correlate with greater optimism regarding the evolution of the pandemic and increased well-being (notably through increased positive affect) [77], both directly related to higher GQOL. In turn, Agreeableness was found to be associated with an advantageous perception of life changes and with a greater chance to receive help in difficult circumstances [78], this decreasing anxiety and Perceived Stress and improving GQOL.

In contrast, Neuroticism was not correlated as an independent predictor of GQOL.

Concerning the state psychological variables:

- Hypothesis 3 was partially verified (Fear of COVID-19 was negatively correlated with QOL only in women). It is a safe assumption that other stressors involved in the male students’ lives during the pandemic may have contributed to the decrease in their GQOL, such as the impact of protective measures on their daily routine, the additional financial struggles experienced in pursuing their studies, the possible academic delays because of home confinement and the ambivalent impact of online learning [41,48]. These factors were probably experienced by women too, but their comparative weight, when considering the higher Fear of COVID-19, was lower.
- Hypothesis 4 was verified (Perceived Stress was negatively correlated with GQOL in both men and women). Due to the close connection between high Perceived Stress and psychiatric comorbidity, this brings attention to academic students as a potential risk group for post-traumatic stress disorder, generalized anxiety major depression, substance abuse and suicide. Although these consequences were not distinctively explored in our study (given its cross-sectional design), they represent a reason for future research exploring the connection between them and the overall stress exposure during the COVID-19 pandemic.

The results of the hierarchical regression analysis suggest that several personality traits (Conscientiousness, Extraversion and Agreeableness) could serve as reliable predictors of GQOL among medical academic students exposed to the consequences of the COVID-19 pandemic. This list is complemented by Perceived Stress, which represents a strong predictor of low GQOL. These associations were independent of the students’ gender, residence and study cycle.

While personality traits are not typically the subject of short- and average-term psychotherapeutic interventions, the important association between Perceived Stress and GQOL suggests several possible ways of mitigating the impact of the pandemic on medical students. These interventions could be centered, for example, on improving the students’ repertoire of coping skills, increasing their stress awareness or engaging them in individual and group discussion or brief interventions aimed to realize the primary or secondary prevention of COVID-19-related stress exposure.

Additionally, several distinct personality characteristics, such as being friendly, sociable and compliant, could represent advantages in handling the challenges brought by the COVID-19 pandemic and its extensive consequences.

### 4.3. Limitations

The transversal character of this research does not allow for strong claims to be put forward regarding causality. The results in our sample have limited generalizability to all students, given the self-selection of study participants. Also, their self-reported answers to the measurement tools may not entirely reflect the real-life experiences. The results could have had higher representativity if the collection of data had been extended nationwide. Given the higher proportion of female participants, our results may be more reflective of female medical students’ experiences and perceptions. Future research should aim to recruit a more gender-balanced sample. While the results provide valuable insights into the socio-demographic GQOL correlates (including the place of residence and internal migration), a more in-depth analysis of multiculturality could have enhanced the generalizability and applicability of the findings to broader populations. The study did not explore the possible mediator role of the participants’ personal or family history with COVID-19 in influencing outcome variables, as the morbidity and mortality due to COVID-19 were largely variable, and the study design was cross-sectional. The respondents were medical students; therefore, their perception of the consequences of a biological risk, such as COVID-19, may have been different from the perception of risk by students from other disciplines outside the health field. The lack of pre-pandemic GQOL reference data in the Romanian medical academic facilities prevents the assessment of the impact of COVID-19 on GQOL in terms of predictions.

## 5. Conclusions

The COVID-19 pandemic brought major and potentially long-lasting changes in GQOL for people all over the globe, necessitating a thorough evaluation of its associated factors and their comparative importance. Our research points out the importance of several personality factors (Conscientiousness, Extraversion, Agreeableness), which are associated with higher GQOL, and, oppositely, the negative association of Perceived Stress. Fear of COVID-19 was not specifically associated with a decrease in GQOL; however, it was significantly encountered in women, making them a potential group at risk, suggesting early prevention and/or psychological intervention. Future research could highlight more specific areas where GQOL is affected. Using a longitudinal design could provide insights into the long-term effects of intensely stressful circumstances, such as the COVID-19 pandemic. Expanding the sample to include diverse healthcare professional groups and conducting comparative studies between them could further improve the generalizability of the findings.

This study brings useful information for a better management of COVID-19 consequences among medical students (these students representing one of the population groups significantly affected by this disease, both in the medical and psychological sense). In this respect, our results could assist in better handling of personality- and lifestyle-related vulnerabilities in this particular group, and they could bring an advantage in the event of a future epidemiological threat similar to COVID-19.

## Figures and Tables

**Table 1:** Descriptive data of socio-demographic variables.

Variable		Statistic Parameter	
*Quantitative*		*Mean*	*Standard Deviation*
Age		21.40	1.749
*Qualitative*		*N*	*%*
Gender	Male	113	18.43
	Female	500	81.57
Study year	First	128	20.88
	Second	202	32.96
	Preclinical (total)	330	53.84
	Third	90	14.68
	Fourth	80	13.05
	Fifth	75	12.23
	Sixth	38	6.20
	Clinical (total)	283	46.16
Kept residence during the pandemic	Yes	380	61.99
	No	233	38.01

**Table 2:** Descriptive data (psychosocial variables).

Variable		Mean	Standard Deviation
Big Five characteristics	Openness	10.89	2.06
	Conscientiousness	10.25	2.64
	Agreeableness	10.14	1.85
	Neuroticism	9.74	2.64
	Extraversion	9.55	2.30
Perceived Stress		20.24	7.13
Fear of COVID-19		14.03	5.12
Global quality of life (GQOL)		26.27	5.51

**Table 3:** Personality dimensions and global quality of life (linear regression) *.

Model	Unstandardized Coefficients		Standardized Coefficients	t	Sig.
	B	Std. Error	Beta		
(Constant)	22.198	2.193		10.122	0.001
Openness	−0.056	0.099	−0.021	−0.561	0.575
Conscientiousness	0.352	0.101	0.135	3.484	0.001
Extraversion	0.401	0.093	0.167	4.319	0.000
Agreeableness	0.273	0.111	0.092	2.464	0.014
Neuroticism	−0.568	0.081	−0.271	−7.031	0.001

* Dependent variable: GQOL; *p* < 0.05.

## Data Availability

Data in anonymized form are available upon reasonable request from study authors.
